# Behavior and body size modulate the defense of toxin-containing sawfly larvae against ants

**DOI:** 10.1038/s41598-021-93074-2

**Published:** 2021-06-30

**Authors:** Jean-Luc Boevé

**Affiliations:** OD Taxonomy and Phylogeny, Royal Belgian Institute of Natural Sciences, Rue Vautier 29, 1000 Brussels, Belgium

**Keywords:** Behavioural methods, Biological techniques, Ecology, Physiology

## Abstract

The sawfly larvae of most Argidae and Pergidae (Hymenoptera: Symphyta) species contain toxic peptides, and these along with other traits contribute to their defense. However, the effectiveness of their defense strategy, especially against ants, remains poorly quantified. Here, five *Arge* species, *A. berberidis*, *A. nigripes*, *A. ochropus*, *A. pagana*, *A. pullata*, plus three Pergidae species, *Lophyrotoma analis*, *Lophyrotoma zonalis*, *Philomastix macleaii*, were tested in laboratory bioassays on ant workers mainly of *Myrmica rubra*. The experiments focused on short-term predator–prey interactions, sawfly survival rate after long-term interactions, and feeding deterrence of the sawfly hemolymph. The larvae of *Arge* species were generally surrounded by few ants, which rarely bit them, whereas larvae of Pergidae, especially *P. macleaii*, had more ants around with more biting. A detailed behavioral analysis of *Arge*-ant interactions revealed that larval body size and abdomen raising behavior were two determinants of ant responses. Another determinant may be the emission of a volatile secretion by non-eversible ventro-abdominal glands. The crude hemolymph of all tested species, the five *Arge* species and *L. zonalis*, was a strong feeding deterrent and remained active at a ten-fold dilution. Furthermore, the study revealed that the taxon-specific behavior of ants, sting or spray, impacted the survival of *A. pagana* but not the large body-sized *A. pullata*. The overall results suggest that the ability of *Arge* and Pergidae larvae to defend against ants is influenced by the body size and behavior of the larvae, as well as by chemicals.

## Introduction

**Figure 1:**
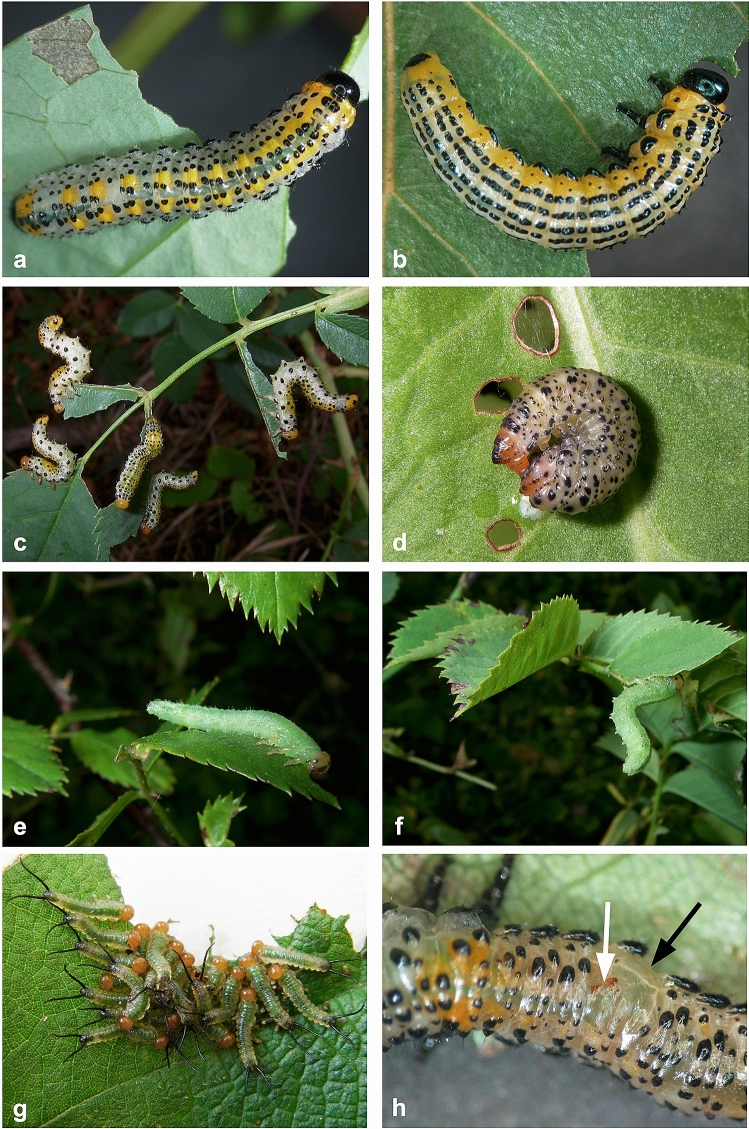
Pictures showing larvae of most of the species used in the study. (**a**) *Arge berberidis*, (**b**) *Arge pullata*, (**c**) *Arge ochropus*, (**d**) *Lophyrotoma analis*, (**e**–**f**) *Arge nigripes*, (**g**) *Philomastix macleaii*, (**h**) *A. ochropus*. (**c**, **f**) Raising the abdomen is a common defensive behavior in *Arge* species. (**h**) Following long-term interactions with ants in laboratory tests, larvae can show swellings (*black arrow*) and/or more or less melanized scars (*white arrow*), here both at the right side of the fourth abdominal segment. For body sizes and complete names of the species, see Table 1. Pictures by Jean-Luc Boevé (**a**–**g**) and Charles-Albert Petre (**h**).

Many insects defend against predation with traits involving their behavior, morphology, physiology and chemistry^1^. Unique peptides found in the larvae of some Argidae and Pergidae (Hymenoptera: Symphyta) are probably used in such defense strategies. These chemical compounds were first revealed in Australia when grazing cattle had ingested larvae of *Lophyrotoma interrupta* (Pergidae) that pullulated around their eucalypt host plant^2–4^. Cases of livestock dying after ingesting sawfly larvae of *Arge pullata* (Argidae; Fig. 1) were observed in Denmark and from ingesting larvae of *Perreyia flavipes* (Pergidae) in South American countries^4–7^. Hepta- and octapeptides were isolated and identified from the sawfly species, and toxicological tests showed that they can kill vertebrates^5,8^. From an eco-evolutionary perspective, however, the toxic peptides that occur commonly among these two sawfly families were admittedly driven not by livestock but other organisms^9,10^.

Besides the toxins, which are at highest concentrations in the hemolymph of an argid and a pergid species^11^, alternative or complementary defensive mechanisms are known in these larvae. The most striking is probably the oral discharge of an oily fluid from a pharyngeal diverticulum found in the Perginae^12–14^, a group of the Pergidae in which toxins have not been detected^9^. Furthermore, taxon-specific organs are sometimes uncertainly related with a chemical constituent, and they may or may not play a defensive role. Examples are the non-eversible ventro-abdominal glands in *Arge* and *Acordulecera*, the flask-shaped latero-abdominal glands in *Atomacera* and *Sterictiphora*, the eversible dorsal glands in *Styracotechys dicelysma*, the caudal filaments in *Lagideus podocarpus*, and the body pubescence^12,15–18^.

Argidae and Pergidae larvae differ in visual appearance and level of gregariousness from one species to another^10^. Differences in appearance can also occur during ontogeny, from one larval instar to another, in Argidae^17,19–21^. In sawfly larvae as in other insects, appearance is expected to be primarily directed against birds by functioning as a visual signal warning of the presence of a defense^22^, whereas gregariousness would influence interactions with vertebrates and invertebrates, because grouped individuals are better chemically defended than single ones^10,23^.

The predators of sawfly larvae are birds as well as invertebrates such as ants and bugs^24,25^. Predatory ants are known to affect sawfly populations^26,27^. Laboratory bioassays show that ethanolic larval extracts of the argids *Arge pagana* and *A. pullata* are a feeding deterrent against ant workers of *Myrmica rubra* (Myrmicinae). Among larval body parts tested, the hemolymph extracts are the most active^17^. Also the oily fluid regurgitated by larvae of several Perginae species is a deterrent when tested on the ants *Iridomyrmex purpureus*, *Myrmecia pilosula* and *Formica exsectoides* as well as other predators^13^. But as far as known, no other Argidae or Pergidae species were tested against ants.

The aim of the present study is to assess the defense effectiveness of five Argidae and three Pergidae species against workers of several ant species. Since the two species, *A. pagana* and *A. pullata*, tested so far against *M. rubra* appear well defended^17^, a more detailed approach was adopted to detect interspecific variations in the defensive effectiveness of sawfly larvae by extending the number of tested prey and predator species. Fresh sawfly hemolymph was also tested on ants, because it contains high concentrations of toxic peptides^11^ and because, more generally, harmful hemolymph is a key determinant in the defense strategy of several sawfly larvae^28^ as well as other insects^29^. The links between defense effectiveness and chemical, morphological, behavioral, and ecological traits of the larvae are discussed.

## Results

Several behaviors were observed in the *Arge* and Pergidae larvae (Table 1) when ants approached, contacted and/or bit them. Such behaviors are usually displayed when the larvae are disturbed (Fig. 1). The behaviors are summarized here, knowing that not all tested sawfly larvae of one taxon showed them. The *Arge* larvae raised their abdomen for several seconds. Larvae of *Lophyrotoma analis* became immobile and/or curled the abdomen more or less around the head. The larva of *Lophyrotoma zonalis* also remained immobile even if attacked by several ants, but it could slightly raise the abdomen. *Lophyrotoma zonalis* possesses a single caudal filament, *Philomastix macleaii* two caudal filaments, and these organs were directed towards approaching ants. If attacked, however, *P. macleaii* made violent and uncoordinated body movements in an attempt to escape ant attacks.

**Table 1:** Data about host plant, geographical distribution, larval appearance, gregariousness level, body size and toxin concentration of the sawfly species used in the study. Geographical distribution data are from the literature^30^; *A. ochropus* has been introduced in the Nearctic. Appearance and gregariousness data are given for last-instar larvae, but bioassays with *A. pagana* and *P. macleaii* also included younger larvae. Gregariousness level: gregarious (i.e., larvae on one leaf or several adjacent leaves) or aggregated (i.e., generally less than three larvae per leaf). Body sizes are from the literature^15^ and own observations; (*) values without considering the caudal appendice(s). Data of toxic peptide concentrations are given as a percentages of fresh weight (% FW) and are from the literature^9,31^. Larvae at instars 5 and 6 (L5-6) or 3 to 5 (L3-5). (–) Unknown. Use in bioassays: (a) short-term interactions, (b) survival after long-term interactions, (c) feeding deterrence of hemolymph. Ant species used: *Myrmica rubra* (Linnaeus, 1758), *Formica polyctena* Förster, 1856, and *Rhytidoponera metallica* (Smith, 1858).

Sawfly species	Field host-plant genus	Geographical distribution	Appearance	Gregariousness	Body size (mm)	Toxic peptides (% FW)	Use in bioassays
**Argidae**							
*Arge berberidis* Schrank, 1802	*Berberis*	Palaearctic	Conspicuous	Gregarious	18	0.12	a, c
*Arge nigripes* (Retzius, 1783)	*Rosa*	West Palaearctic	Cryptic	Aggregated	20	0.15	a, c
*Arge ochropus* (Gmelin, 1790)	*Rosa*	West Palaearctic	Conspicuous	Aggregated	20	–	a, b, c
*Arge pagana* (Panzer, 1797); L5–6	*Rosa*	Palaearctic, Oriental	Conspicuous	Gregarious	20	0.16	a, b, c
*A. pagana*; L3–5	*id.*	*id.*	Cryptic	Gregarious	15	–	a, c
*Arge pullata* (Zaddach, 1859)	*Betula*	Palaearctic	Conspicuous	Gregarious	28	–	a, b, c
**Pergidae**							
*Lophyrotoma analis* (Costa, 1864)	*Rumex*	Australasian	Conspicuous	Aggregated	18	0.37	a
*Lophyrotoma zonalis* (Rohwer, 1910)	*Melaleuca*	Australasian	Conspicuous	Aggregated	23*	0.72	a, b, c
*Philomastix macleaii* (Westwood, 1880); older	*Rubus*	Australasian	Conspicuous	Aggregated	20*	0.41	a
*P. macleaii*; younger	*id.*	*id.*	Conspicuous	Gregarious	13*	–	a

**Figure 2:**
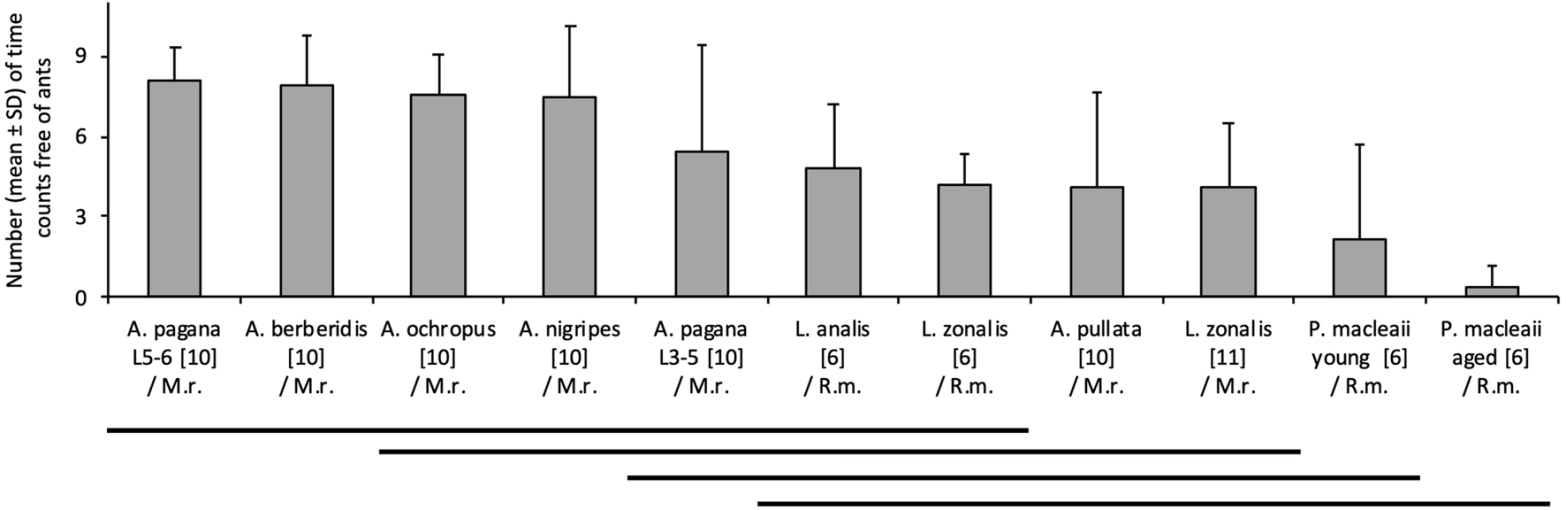
Defense effectiveness of sawfly larvae/instars against ants in short-term interactions. The histogram represents the numbers of time counts with no ant contacting a larva; the values are given as mean and standard deviation. Sawfly species/instars are given on the *X*-axis (Table 1). They are ordered from left to right by decreasing time-counts free of ants. The number of tested larvae is given between square brackets. The ant species used are *Myrmica rubra* (M.r.) and *Rhytidoponera metallica* (R.m.). Pairs of means grouped by a horizontal line are not significantly different from each other (Tukey–Kramer method, *P* > 0.05).

Among the *Arge* species/instars tested against *M. rubra*, *A. pullata* was more often attacked than the other species (Fig. 2). Among the Pergidae, *P. macleaii* and especially its aged larvae, were more often attacked than *Lophyrotoma* spp. *Lophyrotoma zonalis* was tested against *M. rubra* as well as *Rhytidoponera metallica* (Ectatomminae), and it was attacked by these two ant species at similar frequencies (Fig. 2).

**Figure 3:**
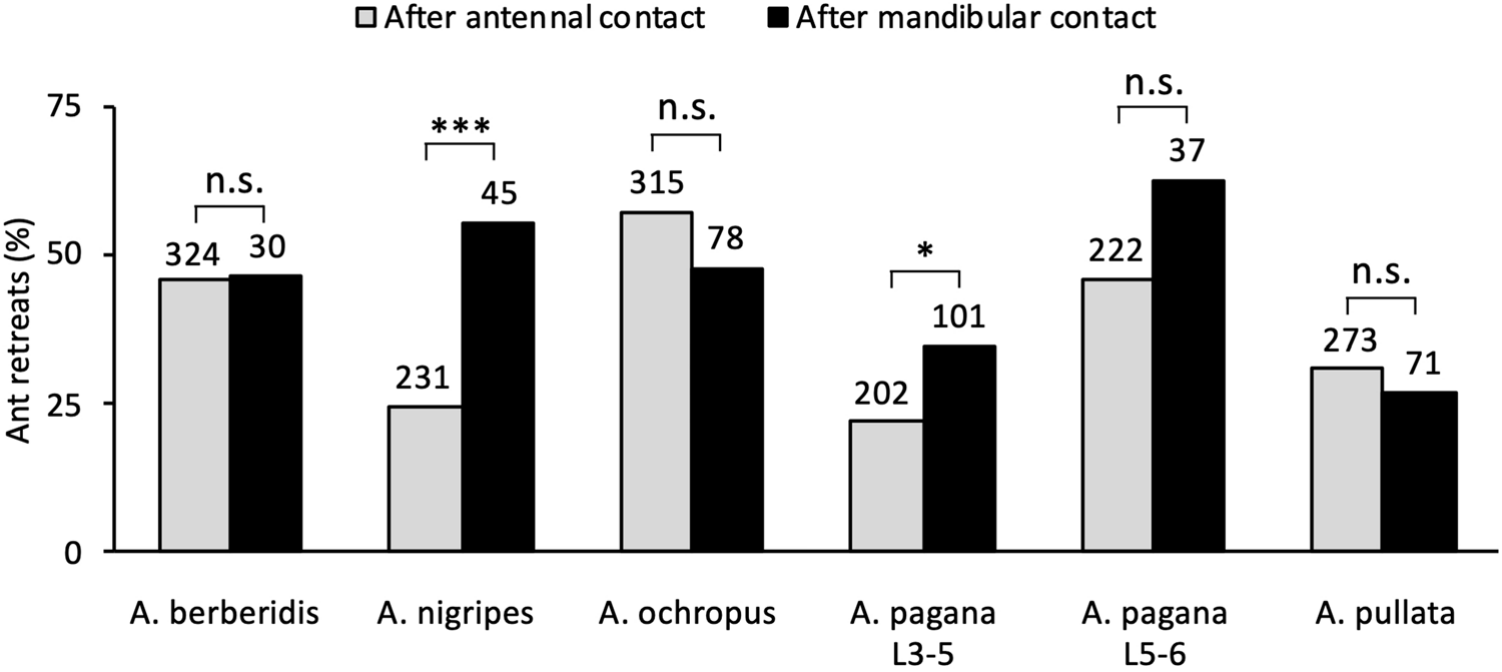
Ant retreating from a sawfly larva in short-term interactions. The histogram represents percentages of ant retreats after contacting a larva with its antennae or mandibles. Sawfly species/instars are given on the *X*-axis (Table 1). Above each histogram bar is given the total number of antennal or mandibular contacts. Statistical results were obtained by Fisher exact probability tests, two-tailed, comparing the number of retreats after an ant’s antennal vs. mandibular contact: *P* > 0.05 (n.s.), *P* < 0.05 (*), *P* < 0.001 (***).

A significantly higher percentage of ants retreated after a mandibular contact than after an antennal contact when interacting with *Arge nigripes* and *A. pagana* L3–5, whereas the percentages were similar when ants interacted with the other *Arge* species/instars (Fig. 3).

Ants could approach a larva without contacting it with its antennae or mandibles. Although *Arge berberidis*, *Arge ochropus* and *A. pullata* larvae did not raise their abdomen at the approach, most ants retreated nevertheless (Table 2). Once contacted by an ant, the larva could either not react or raise its abdomen, the ant’s response to either behavior was to show a neutral response or to retreat. The association between larval and ant reactions were as follows. For interactions starting with an ant antennal contact, the fact that the larvae of *A. berberidis*, *A. nigripes*, *A. pagana* L5–6 and *A. pullata* did not react resulted in less ants retreating than showing neutral reactions, whereas larvae raising their abdomen resulted in proportionally more ant retreats than neutral reactions; the associations were not significant with larvae of *A. ochropus* and *A. pagana* L3–5 (Table 2). Following ant mandibular contacts, the associations were significant with the larvae of *A. nigripes*, *A. ochropus* and *A. pagana* L5–6, but not with the other *Arge* species/instars (Table 2). The associations were never significant following ant bites, which occurred rarely, maximum numbers being 11 bites in *A. pagana* L3–5 and 10 bites in *A. pullata* (Table 2).

**Table 2:** Behavioral transitions of ant-larva-ant events between *Myrmica rubra* ants and an *Arge* sawfly larva in short-term interactions. The interaction frequencies are described by following three successive behaviors. First, the ant either approached the larva without contacting it, had an antennal contact, had mandibular contact, or bit it. Second, the larva had its abdomen either raised, or not. Third, the ant could either retreat, or not (i.e., neutral response). Sawfly species/instars (see Table 1) are: *A. berberidis* (A.be.), *A. nigripes* (A.ni.), *A. ochropus* (A.oc.), *A. pagana* (A.pa. L3-5 and L5-6), and *A. pullata* (A.pu.). Statistical results were obtained by Fisher exact probability tests (*P*, two-tailed), comparing the number of times a larva raising or not its abdomen was followed by an ant’s neutral response or retreat. *P* > 0.05 (n.s.).

Ant > Larva > Ant	A.be	A.ni	A.oc	A.pa. L3-5	A.pa. L5-6	A.pu
no contact > no reaction > neutral response	2	0	8	1	3	0
no contact > no reaction > retreat	72	3	69	0	8	50
no contact > abdomen raise > neutral response	0	0	0	0	0	0
no contact > abdomen raise > retreat	1	9	6	0	5	2
*P*	n.s.	n.s.	n.s.	n.s.	n.s.	n.s.
antennal contact > no reaction > neutral response	173	146	124	97	99	173
antennal contact > no reaction > retreat	125	32	162	27	56	67
antennal contact > abdomen raise > neutral response	3	28	11	60	22	15
antennal contact > abdomen raise > retreat	23	25	18	18	45	18
*P*	< 0.001	< 0.001	n.s.	n.s.	< 0.001	0.003
mandibular contact > no reaction > neutral response	15	14	37	40	10	49
mandibular contact > no reaction > retreat	9	6	26	14	6	15
mandibular contact > abdomen raise > neutral response	1	6	4	26	4	3
mandibular contact > abdomen raise > retreat	5	19	11	21	17	4
*P*	n.s.	0.003	0.042	n.s.	0.015	n.s.
bite > no reaction > neutral response	0	1	0	0	1	6
bite > no reaction > retreat	1	1	1	0	0	0
bite > abdomen raise > neutral response	0	0	0	4	0	2
bite > abdomen raise > retreat	1	3	1	7	0	2
*P*	n.s.	n.s.	n.s.	n.s.	n.s.	n.s.

The survival of Pergidae larvae tested (six individuals per species and age; 5-min interactions) against *R. metallica* was as follows: all *L. analis* and *L. zonalis* survived, whereas all young and aged *P. macleaii* died. The lowest percentage of non-injured larvae at the end of the 24-h period of sawfly-ant interactions was reached by *A. ochropus* (with 17% intact larvae) against *Formica polyctena* (Formicinae), the highest one being reached by *A. pagana* L5–6 (42%) against *M. rubra* (Table 3). Two typical wounds were noticed in the larvae that were injured, either scars only, or scars and swellings (Fig. 1h). Scars only were observed on larvae that were confronted to *M. rubra* ants, whereas scars and swellings were related to interactions with *F. polyctena* (Table 3).

**Table 3:** Health state and survival of sawfly larvae following long-term interactions with ants of *Myrmica rubra* and *Rhytidoponera metallica*. Sawfly-ant interactions lasted for 24 h, i.e. from t = 0 to 1 day. Number of experiment repetitions (N). The percentage of intact larvae is given, based on the health state of the larvae that was recorded at t = 1 day; intact larvae demonstrated no visible signs of scars and swellings. The survival of the larvae was recorded at t = 1 day and 3 days. (L5-6) Larvae at instars 5 and 6. (–) Not recorded.

Sawfly species	Ant species	N	Intact larvae (%)	Larvae (%) with ≥ 1 scar(s)	Larvae (%) with ≥ 1 swelling(s)	Survival (%) at 1 day	Survival (%) at 3 days
*Arge pagana* L5–6	*M. rubra*	12	42	58	0	100	42
*Arge pullata*	*M. rubra*	12	25	75	0	100	67
*Lophyrotoma zonalis*	*M. rubra*	12	–	–	–	100	100
*Arge pagana* L5–6	*F. polyctena*	18	33	67	50	100	94
*Arge ochropus*	*F. polyctena*	18	17	83	50	94	78
*Arge pullata*	*F. polyctena*	18	22	78	22	94	67

At least 67% of larvae were alive at t = 1 day (the end of the sawfly-ant interactions) and still alive at t = 3 days, except for *A. pagana* L5–6 against *M. rubra* (Table 3). *Arge pagana* L5–6 and *A. pullata* larvae were both tested on ants of *M. rubra* and *F. polyctena*. The survival rate of *A. pagana* at t = 3 days was significantly higher with *F. polyctena* than *M. rubra* (*P* < 0.001, Fisher exact probability test), whereas the survival rate of *A. pullata* was independent of the ant species used (*P* > 0.05; Table 3).

All undiluted hemolymph samples tested on *M. rubra* reached deterrence rates of 85% to 100% (Table 4). The deterrence rate of the 1:10 dilution was significant for every tested species/instar. From the undiluted hemolymph to the 1:10 dilution, the deterrence rate significantly decreased only for *A. nigripes* and *A. pullata*. Comparing 1:10 with 1:100 dilutions, the deterrence rate significantly decreased in all tested sawfly species/instars. At the 1:100 dilution, the deterrence rate was significant with the hemolymph from *A. ochropus*, *A. pagana* (both L3–5 and L5–6) and *L. zonalis*, but non-significant with the hemolymph from *A. berberidis*, *A. nigripes* and *A. pullata* (Table 4).

**Table 4:** Deterrence rates of crude hemolymph from sawfly larvae tested at three concentrations on ant *Myrmica rubra*. Deterrence of the hemolymph was compared with the control. Hemolymph was used at three concentrations (Conc.). Larvae at instars 3 to 5 (L3-5) or 5 and 6 (L5-6). Each test was repeated 12 times, except for one sample with five repetitions (#5). Data for *A. pagana* L5–6 are from Petre et al. (2007)^17^. Values at each concentration are given as percentages of deterrence rate, with total numbers of ants on hemolymph vs. control droplets given between parentheses. Statistical results under each concentration are from Wilcoxon signed-rank tests, two-tailed: *P* > 0.05 (n.s.), *P* < 0.05 (*), *P* < 0.01 (**). Statistical results between two table columns compare the data from these columns, by Fisher exact probability tests, two-tailed: *P* > 0.05 (n.s.).

Species	Conc. 1	*P*	Conc. 1:10	*P*	Conc. 1:100
*Arge berberidis*	89** (2/33)	n.s.	89** (3/54)	< 0.001	10^n.s.^ (38/46)
*Arge nigripes*	94** (2/63)	0.012	66** (9/44)	< 0.001	-2^n.s.^ (43/40)
*Arge ochropus*	97** (1/65)	n.s.	84** (6/70)	< 0.001	34* (34/69)
*Arge pagana* L3–5	85^n.s.^ (1/12) #5	n.s.	90** (4/73)	< 0.001	36* (15/32)
*Arge pagana* L5–6	95** (2/73)	n.s.	94** (2/66)	< 0.001	33** (32/63)
*Arge pullata*	100** (0/81)	< 0.001	69** (10/55)	< 0.001	6^n.s.^ (49/55)
*Lophyrotoma zonalis*	95** (2/76)	n.s.	100** (0/28)	< 0.001	30* (20/37)

**Figure 4:**
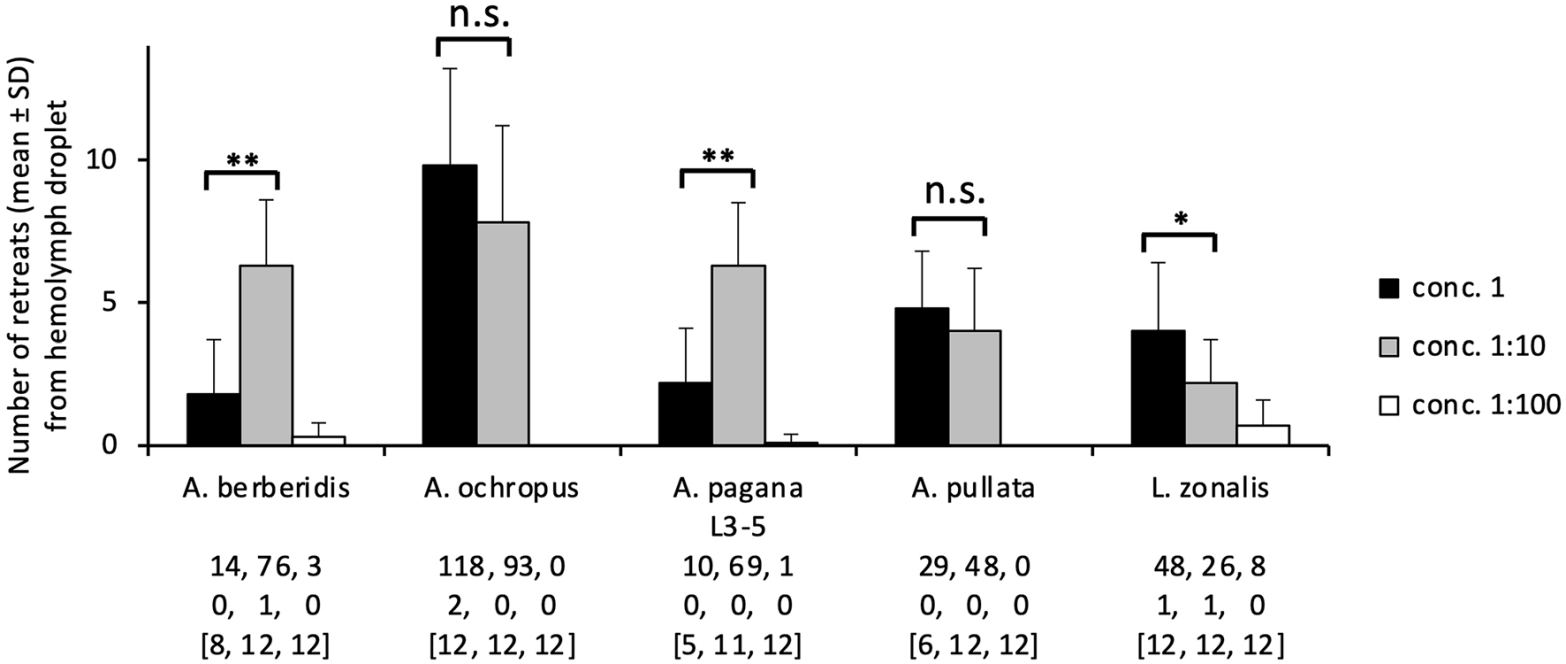
Retreats of *Myrmica rubra* ants from sawfly hemolymph droplets tested at three concentrations. The histogram represents the number of ant retreats from undiluted and diluted hemolymph droplets, with values given as mean and standard deviation. Statistical results above histogram bars are from Mann–Whitney tests, two tailed, by comparing the data of concentrations 1 vs. 1:10: *P* > 0.05 (n.s.), *P* < 0.05 (*), *P* < 0.01 (**). Sawfly species/instars are given on the *X*-axis (Table 1). Below these names and per concentration, the absolute numbers of retreats from the test droplets (*first row*) and control droplets (*second row*) are given as well as the number of test repetitions between square brackets.

By counting the number of ants walking backwards, it was determined that significantly more ants retreated from the crude hemolymph than the water droplet control (*P* < 0.001, five times, Binomial tests; for *A. berberidis*, *A. ochropus*, *A. pagana* L3–5, *A. pullata*, *L. zonalis*; Fig. 4). This difference in the number of ant retreats remained significant when testing the hemolymph 1:10 dilution of all species (*P* < 0.001, five times), but this difference was significant only when testing the 1:100 hemolymph dilution of *L. zonalis* (*P* < 0.005). The number of ants retreating from the hemolymph droplet, averaged over the number of tests performed, was significantly higher for the 1:10 diluted hemolymph than the undiluted one for *A. berberidis* and *A. pagana* L3–5; these numbers were similar for *A. ochropus* and *A. pullata*, and they were lower for *L. zonalis* (Fig. 4).

## Discussion

Predatory ants can cause high mortality rates among arthropods^32–37^, which results in part from their diversity and ubiquity in most terrestrial ecosystems^38–41^. Conversely, prey species exhibit a diversity of anti-ant substances that can be emitted at any life stage^29,42–44^ and present in their by-product such as feces and silk^45,46^. Defensive compounds are generally not target specific within a studied predator–prey system since they often act against ants as well as birds, and they can also possess antimicrobial and antifungal activities^47,48^. But some insect species contain chemicals that are target specific towards guilds of natural enemies, acting for instance on predators but not parasitoids^49^, or even towards predator types by acting on ants but not birds, or vice-versa^50^. Thus, this study, which pits a prey group against one predator type, constitutes a simplified situation compared with prey species facing multiple and various antagonists in natural conditions.

Nevertheless, comparing different prey species tested identically against a predator allows for the detection of a defense effectiveness that can potentially vary across the prey species. The laboratory bioassays focusing here on short-term, predator–prey interactions revealed that sawfly larvae were generally well defended against ant attacks. The larvae of *Arge* were generally not surrounded by ants and, even when they were, few ant bites were observed. This contrasts with sawfly-ant systems where some sawfly species are well defended whereas others are not, although being closely related and/or feeding on a same host plant^28,51,52^. In these systems, differences in defensive effectiveness are explained by distinct morphological or physiological adaptations across taxa, whereas all *Arge* species analyzed contain toxic peptides^9,31^ and their hemolymph is a strong feeding deterrent that may cause ant paralysis^17^. The hemolymph of *A. pagana* and *L. zonalis* contains more toxic peptides than their other organs^11^. Concentrations of toxic peptides remain nearly constant across larval instars in *A. berberidis*^31^. Moreover, three argid and pergid species were shown to be well defended against attacks by bugs^53^ that typically pierce the prey's integument to feed on internal organs. Caution is however required in explaining feeding deterrence because it may be due to the toxins but also to other compounds with harmful effects such as entanglement. The counterintuitive results that a lower concentration of hemolymph from *A. berberidis* and *A. pagana* L3–5 resulted in more retreats than the undiluted hemolymph (Fig. 4) suggest that the deterrent effect is more complex than a simple correlation between concentration and measured effectiveness. Some explanations may include the possibility that the undiluted hemolymph was so heavily loaded with distasteful compounds that few ants even started to feed, and/or that ants were paralyzed especially while feeding on the droplet of undiluted hemolymph, which prevented from removing their body from the droplet.

There were proportionately more ant retreats than neutral responses when the larva raised its abdomen after antennal and mandibular contacts in all tested *Arge* species, while *A. pagana* L3–5 was the only case in which raising the abdomen never influenced ant response. This might be due to their small body size which made them physically unable to impede further attacks. Arthropod predators prefer smaller than larger prey^54^. Relatively few *A. pullata* larvae were free of ants. It was the largest *Arge* species tested. Since a high ant density was used in the bioassay, sawfly-ant contacts may have been less avoidable especially with these large larvae.

More generally the question is whether or not it was advantageous for the sawfly larvae to raise their abdomen. One function can be to mechanically dislodge biting ants by crushing them with the tip of the abdomen^55^. Another function might be its involvement in chemical defense. The larvae of the Nematinae (Tenthredinidae) possess eversible ventro-abdominal glands that can emit a volatile repellent secretion^56^, and raising the abdomen often constitutes a first behavioral step before turning the glands inside-out^51^. In Argidae, ventral glands also exist, but these glands are non-eversible and the chemistry of the emitted volatiles remains largely unexplored^12,17^. The bioassays revealed that ants can retreat when a larva raises its abdomen, adding to evidence that volatiles play a defensive role^17^. Still another function is the visual impact of the body movements on predators hunting by sight and that can be frightened at distance^22^, especially when gregarious insects simultaneously raise their abdomens. Argidae and Pergidae larvae are commonly aggregated or truly gregarious, and grouped individuals are better defended than single ones against vertebrate and invertebrate predators^10^.

After long-term interactions with ants, more *A. pagana* L5–6 survived when facing *F. polyctena* than *M. rubra*, whereas survival of *A. pullata* was similar against the two ant species. Attack behaviors known in ants include biting, stinging and/or spraying irritant compounds. Note that this chemo-behavioral arsenal can play offensive as well as defensive roles. Myrmicinae ants such as *M. rubra* can bite and sting, whereas Formicinae ants such as *F. polyctena* can bite and spray^57^. It is probable that these specific ant behaviors produced the symptoms observed on the sawfly larvae: bites caused scars, spraying by *F. polyctena* caused swellings. But no stings were observed on the larval body surface although *M. rubra* almost certainly stung the larvae, and one can assume a sting to be more lethal than spraying. This would explain the low survival of *A. pagana* attacked by *M. rubra*, while the large body size of *A. pullata* would explain why the two ant species had a similar impact on the survival of this sawfly species.

Compared with the Argidae, the Pergidae tended to be contacted more often by ants and this was especially the case with aged larvae of *P. macleaii*. Both young and aged larvae of this species did not survive the shot-term interaction tests (lasting only 5 min), probably because their vigorous whole-body movements stimulated the ants to bite. In contrast, the immobility of the two tested *Lophyrotoma* species tended to impede the ant attacks. Becoming immobile is a defensive behavior known in some Tenthredinidae species^58^. *Philomastix macleaii* as well as the pergid *L. podocarpus* possess two caudal appendices; these organs play a defensive role in the latter species when a larva is approached by only a few ants^18^. *Lophyrotoma zonalis* larvae were tested on *M. rubra* and *R. metallica*, yielding similar numbers of ant-free time counts. This suggests that the defensive effectiveness of these sawfly larvae is independent of which ant species is attacking them.

The bioassays evidenced that body size and abdomen raising behavior were important determinants of the defensive effectiveness in Argidae and Pergidae larvae. Additional study is necessary to fully understand why defense was generally more effective for *Arge* than for Pergidae larvae.

## Methods

### Study system

The sawfly larvae were collected in the field in Belgium and Australia, and identified using published keys^15,59,60^ and reference material. To illustrate the habitus and some behaviors of the larvae, pictures were taken in the field, using a camera Pentax Optio W10, and in the laboratory with a Canon PowerShot G3. The larvae were kept in boxes for several days on fresh host-plant leaves, until their use in laboratory bioassays (Table 1).

Workers of the following ant species were used: *M. rubra*, *F. polyctena*, and *R. metallica*. *Myrmica rubra* and sawfly larvae can be sympatric (personal observation). This ant species and *F. polyctena* are generalists feeding on carbohydrate and protein sources, *F. polyctena* feeding among others on sawfly larvae^61^. *Rhytidoponera metallica* is known as a seed disperser but is also preying on insects^62^. Ant colonies in whole or part were collected in the field, the ants maintained in the laboratory by feeding them ad libitum with a sugar solution and thawed cockroaches. Not all sawfly species could be tested on every ant species and combinations were limited by sawfly availability in the field. For *A. pagana* we compared the conspicuous larval instars 5 and 6 (L5–6) with the cryptic instars, L3–5 (see^17^ for description of instar variations). The younger pergid larvae of *Philomastix macleaii* (mean ± standard deviation: 39 ± 9 mg fresh weight) were compared to older (113 ± 18 mg) larvae.

### Short-term interactions

A single larva was placed for 5 min (t = 0 to 5 min) in a 10 × 10 cm open box containing 20 ant workers of *M. rubra* or 10 of *R. metallica*. The predator–prey interactions were video recorded from t = 2 to 5 min, using a Canon PowerShot G3 camera placed above the open box. This procedure was replicated from 6 to 11 times depending on the species or instar used in the trial. The exact number of replications for each trial is given in Fig. 2. At every 20 s from t = 140 s to 5 min, the event of a larva not contacted by any ant was recorded from the videos, and these non-contact moments were summed over the nine observation times. This avoids calculation biases due to temporal auto-replicated data.

All interactions involving *Arge* larvae against *M. rubra* were further characterized by counting throughout the three-min videos the successive behavioral events of the ants (four possible events), the larva (two), and the ants (two), as follows. The ants either came within less than 1 cm of a larva but without contacting it, they contacted the larva with its antennae, mandibles (i.e., mandibles or anterior part of the head), or bit it. The three first events were clearly discernable, less so a contact with mandibles vs. a bite. After each of these four events, the larva either raised or did not raise its abdomen. Eventually, the ants clearly retreated possibly by running away (“retreat”), or continued their usual patrolling (“neutral response”).

Although the bioassay lasted 5 min, the survival of the larvae was noted 24 h after the Pergidae larvae were tested against *R. metallica*. Survival of the Argidae larvae tested against *M. rubra* was not noted.

### Long-term interactions

A host-plant leaf with a single settled sawfly larva was placed in a cylindrical container (diameter: 3.5 cm, height: 7 cm) with moistened plaster bottom. Ten ants of *M. rubra* or four of *F. polyctena* were added. Larva and ants were left together for 24 h, from t = 0 to 1 day. The health of the larva was recorded at t = 1 day, and its survival at t = 1 day and 3 days. The experiment was replicated 12 times with *M. rubra* and 18 times with *F. polyctena*, always using new larvae and ants. Larvae were defined as unhealthy when at least one scar or swelling was visible on their body.

### Feeding deterrence of hemolymph

Twenty ants of *M. rubra* were placed in a 9-cm diameter Petri dish and starved for 15 h, then deprived of both food and water for an additional 3 h. The integument of live sawfly larvae was pierced to collect hemolymph with a calibrated glass micropipette (Brand GmbH, Wertheim, Germany). At t = 0, two 4-µl drops spaced 3.5 cm apart were placed in the Petri dish: one drop of hemolymph from a single larva vs. one drop of charcoal-filtered tap water. The number of ants feeding on each droplet was counted at t = 3 min. The experiment was replicated with a maximum of 12 larvae, by always using different ants. The crude hemolymph from the larvae was pooled, diluted in charcoal-filtered tap water, and then tested in the same conditions. A deterrence rate was calculated by dividing (C − T) by (C + T), where C and T are the total number of ants feeding on the control and test droplets, respectively.

In a second set of bioassays using the same experimental setup and involving most of the same sawfly species/instars, the number of ants walking at least 1 cm backwards from the hemolymph and control droplets was counted from t = 0 to 4 min.

## Ethical approval

All applicable institutional and/or national guidelines for the care and use of animals were followed.

## Data Availability

The datasets generated and analyzed during the current study are available from the author on reasonable request.
